# Detection of Coccolithophore Blooms With BioGeoChemical‐Argo Floats

**DOI:** 10.1029/2020GL090559

**Published:** 2020-11-25

**Authors:** L. Terrats, H. Claustre, M. Cornec, A. Mangin, G. Neukermans

**Affiliations:** ^1^ Sorbonne Université, CNRS, Laboratoire d'Océanographie de Villefranche, LOV Villefranche‐sur‐Mer France; ^2^ ACRI‐ST Sophia Antipolis France; ^3^ Biology Department, MarSens Research Group Ghent University Ghent Belgium; ^4^ Flanders Marine Institute (VLIZ), InnovOcean site Ostend Belgium

**Keywords:** coccolithophores, *Emiliania huxleyi*, bloom detection, BGC‐Argo floats, bio‐optics, global ocean

## Abstract

Coccolithophores (calcifying phytoplankton) form extensive blooms in temperate and subpolar oceans as evidenced from ocean‐color satellites. This study examines the potential to detect coccolithophore blooms with BioGeoChemical‐Argo (BGC‐Argo) floats, autonomous ocean profilers equipped with bio‐optical and physicochemical sensors. We first matched float data to ocean‐color satellite data of calcite concentration to select floats that sampled coccolithophore blooms. We identified two floats in the Southern Ocean, which measured the particulate beam attenuation coefficient (*c*_p_) in addition to two core BGC‐Argo variables, Chlorophyll‐*a* concentration ([Chl‐*a*]) and the particle backscattering coefficient (*b*_bp_). We show that coccolithophore blooms can be identified from floats by distinctively high values of (1) the *b*_bp_/*c*_p_ ratio, a proxy for the refractive index of suspended particles, and (2) the *b*_bp_/[Chl‐*a*] ratio, measurable by any BGC‐Argo float. The latter thus paves the way to global investigations of environmental control of coccolithophore blooms and their role in carbon export.

## Key Points

- We matched profiling float trajectories with ocean‐color satellite observations of coccolithophore blooms
- Two simple bio‐optical indices permitted successful identification of coccolithophore blooms from floats in the Southern Ocean
- A method for identifying coccolithophore blooms at the global scale is proposed using regional thresholds of bio‐optical float measurements

## 1 Introduction

Detecting major phytoplankton groups is essential to improve our understanding of the global biogeochemical cycles. One such group is the coccolithophores, calcifying phytoplankton which form calcite platelets called coccoliths. The coccolithophore species *Emiliania huxleyi* forms extensive blooms in the temperate and subpolar surface ocean, characterized by detachment and overproduction of coccoliths in the bloom decline phase (Balch et al., 1991, 1996). The accumulation of these high‐refractive‐index calcite particles increases the light backscattered from the ocean surface and colors the water milky‐turquoise (Holligan et al., 1983).

Ocean‐color satellites can detect these milky‐turquoise coccolithophore bloom waters, and remote sensing algorithms have been set up to quantify the associated concentration of particulate inorganic carbon ([PIC] in mmol m^−3^) (Balch et al., 2005; Gordon et al., 2001). Remotely sensed [PIC] has been used to investigate regional, seasonal, and interannual variabilities of coccolithophore blooms at global scale (Hopkins et al., 2015, 2019) and to locate coccolithophore blooms to guide ship‐based sampling (Garcia et al., 2011). Ship‐based measurements of coccolithophore blooms are valuable to calibrate satellite algorithms and investigate the influence of coccolithophore blooms on ocean biogeochemistry (Balch et al., 2005, 2016; Garcia et al., 2011). However, ship missions cover limited spatiotemporal scales and satellites are limited to surface waters and clear‐sky conditions, creating observational gaps, particularly in high‐latitude regions.

BioGeoChemical‐Argo (BGC‐Argo) floats, free‐drifting autonomous ocean profilers equipped with bio‐optical and physicochemical sensors, present an increasingly attractive platform to study environmental control and biogeochemical processes related to phytoplankton blooms (Briggs et al., 2020; Lacour et al., 2015; Mignot et al., 2018). They provide observations at unparalleled temporal and vertical scales and fill observational gaps in undersampled regions, such as high‐latitude waters (Claustre et al., 2020). Among the core variables measured by BGC‐Argo floats are the particulate backscattering coefficient (*b*_bp_, in m^−1^), a proxy for suspended particle concentration, and Chlorophyll‐a fluorescence (FChl‐*a*) from which one can derive the concentration of Chlorophyll‐a ([Chl‐*a*] in mg m^−3^), the primary photosynthetic pigment in phytoplankton cells. Additionally, few floats are equipped with a beam transmissometer, which measures the particulate beam attenuation coefficient (*c*_p_, in m^−1^), another proxy for suspended particle concentration.

The objective of this study is to examine if bio‐optical measurements from BGC‐Argo floats can be used to detect coccolithophore blooms. A first candidate optical property is the *b*_bp_/*c*_p_ ratio, a proxy for the bulk refractive index of suspended particles (Twardowski et al., 2001). Indeed, ship‐based studies have shown that the decline phase of *E. huxleyi* blooms is characterized by large concentrations of shed coccoliths (Balch et al., 1991), non‐chlorophyllous, high‐refractive‐index particles, resulting in increased *b*_bp_/*c*_p_ (Garcia et al., 2011). However, only a limited number of floats measure *c*_p_. Therefore, we also examine the potential to identify blooms from a second candidate property, the *b*_bp_/[Chl‐*a*] ratio, which can be measured by any BGC‐Argo float.

## 2 Materials and Methods

We focused on BGC‐Argo float trajectories in temperate and subpolar open‐ocean waters (i.e., >35°N and >35°S) during the coccolithophore growing seasons (i.e., spring and summer). We matched each profile of the floats with concurrent ocean‐color satellite data. Using the satellite [PIC] time series at each profile location, we set up a technique to decide if the profile was located in a coccolithophore bloom or not and then tested for significant differences in the *b*_bp_/*c*_p_ ratio and the *b*_bp_/[Chl‐*a*] ratio between profiles in coccolithophore bloom and non‐bloom conditions.

### 2.1 Float Measurements and Derived Variables

We first searched the BGC‐Argo float database for floats equipped with a backscattering and chlorophyll‐*a* fluorescence sensor, as well as a beam transmissometer. We identified two floats drifting in the subpolar Southern Ocean. They operated at a daily frequency and were identified by their World Meteorological Organization numbers (WMO) as 6901583 and 6902738. Afterward, we expanded our analyses to every float equipped with backscattering and chlorophyll‐*a* fluorescence sensors.

The ECO Triplet (Three Channel Sensor; WET Labs, Inc., USA) measures FChl‐*a* and the fluorescence of Colored Dissolved Organic Matter (CDOM) at excitation/emission wavelengths of 470/695 and 370/460 nm, respectively, and the angular scattering coefficient of particles at 700 nm and an angle of 124°, from which we derived *b*_bp_ at 700 nm (Boss & Pegau, 2001; Schmechtig et al., 2018). We corrected *b*_bp_ and FChl‐*a* profiles for out‐of‐range values and the sensor drift over time following quality control procedures (Bellacicco et al., 2019). The C‐Rover beam transmissometer (WET Labs, Inc., USA) measures the attenuation of light by particles at 660 nm (*c*_p_ in m^−1^), which is almost entirely attributable to light scattering by particles at that wavelength (Loisel & Morel, 1998). The vertical position of the transmissometer causes sinking material to accumulate progressively on the detection window over time, resulting in a drift of *c*_p_ values (Bishop & Wood, 2009). The drift was corrected by aligning *c*_p_ vertical profiles such that *b*_bp_/*c*_p_ equalled 0.02 at 1000 m. Our sensitivity analysis showed that c_p_ values at the surface did not vary significantly when the ratio *b*_bp_/*c*_p_ ranged from 0.01 to 0.03 at 1,000 m (see Table S1 and Text S1 in the supporting information).

The bio‐optical values transiently increase when particle aggregations or mesopelagic organisms pass in front of the detector, which creates spikes in bio‐optical profiles (Briggs et al., 2011; Haëntjens et al., 2020). We removed these spikes by smoothing the bio‐optical profiles using a 5‐point moving median filter followed by a 7‐point moving average filter (Briggs et al., 2011). Finally, we visually inspected the profiles on a case‐by‐case basis.

We converted FChl‐*a* to [Chl‐*a*] as follows: (i) FChl‐*a* was corrected for the Non‐Photochemical Quenching (NPQ), a photo‐protection mechanism that depresses the FChl‐*a* per unit of [Chl‐*a*] (or *b*_bp_) with diurnal increases in irradiance, through a modification of the method of Xing et al. (2018) (see Text S2 and Figures S1 and S2 in the supporting information for further details on this method), (ii) then converted to [Chl‐*a*] with the factory calibration coefficient, (iii) and finally adjusted with satellite [Chl‐*a*] to compensate for spatial variations in the calibration coefficient revealed by Roesler et al. (2017) (see Text S2 in the supporting information).

We estimated the mixed layer depth (MLD) with the algorithm of Holte and Talley (2009) that relies on temperature, salinity, and density profiles. This method considers physical features, such as the vertical density‐compensation, and provides a more accurate estimate of the MLD in high‐latitude waters compared to threshold and gradient approaches (Holte et al., 2017; Holte & Talley, 2009).

### 2.2 Satellite Data

Daily‐merged ocean‐color satellite data were downloaded from the GlobColour project (ftp://ftp.hermes.acri.fr) with a spatial resolution of 4 km. We described the dynamic of coccolithophore blooms with the [PIC] satellite product using the state‐of‐the‐art retrieval algorithm of Mitchell et al. (2017) (see Figure 8c in Mitchell et al., 2017). We further use %PIC defined as follows:

(1) $$\%\mathrm{PIC}=\frac{\left[\mathrm{PIC}\right]}{\left[\mathrm{PIC}\right]+\left[\mathrm{POC}\right]}\times 100,$$

where [*POC*] is the concentration of particulate organic carbon using the retrieval algorithm of Stramski et al. (2008). In coccolithophore blooms, we appraised the reliability of the band‐difference algorithm of Mitchell et al. (2017) to perceive changes in *b*_bp_ by comparing [*PIC*] to the satellite *b*_bp_ values from the inverse model of Maritorena and Siegel (2005). Finally, we reported the GlobColour [Chl‐a] product which is derived from various algorithms optimized for different water types (i.e., Antoine & Morel, 1996; Gohin et al., 2002; Hu et al., 2012; O'Reilly et al., 1998, 2000), and we removed the estimates in coccolithophore blooms where ocean‐color satellite retrievals of [Chl‐*a*] are biased (Balch et al., 1989).

### 2.3 Matchups Between Satellite and Float Data

The thickness of the surface ocean layer detected by satellites can be derived from vertical profiles of downward irradiance acquired by BGC‐Argo floats. Unfortunately, 67% of irradiance profiles of floats 6901583 and 6902738 were categorized as “probably bad” quality records following the criteria of Organelli et al. (2016) and failed to meet the quality standards for radiometric applications. Therefore, we defined the surface layer sensed by ocean‐color satellites as the first 15 m.

Bailey and Werdell (2006) defined matchup criteria between satellite and in situ data for the sake of validating ocean‐color satellite radiometric products, which requires that the spatial and temporal difference in acquisition be as small as possible (5 × 5 pixel box at native resolution, narrow time window of <3 h). Here, the purpose of matching satellite and in situ data was to obtain a continuous gap‐free time series of satellite data corresponding to the float trajectory. In persistently cloudy areas such as the Southern Ocean, this implies data averaging over larger spatial and temporal scales until a gap‐free time series is achieved (e.g., Haëntjens et al., 2017). Our analyses showed that the optimal configuration to achieve a continuous time series of ocean‐color satellite data matched to float data was a 9‐day average of a 5 × 5 pixel box of 4 km resolution GlobColour satellite products (Figure S4 and Text S4 in the supporting information). In this configuration, the spatiotemporal variability of satellite‐derived [PIC] was within 10 ± 12%, indicating acceptable heterogeneity.

### 2.4 Detection of Coccolithophore Blooms With Satellite Data

To determine if a profile was located in a coccolithophore bloom, we extracted the time series of satellite [PIC] at each profile location. The bloom period was then defined using the algorithm of Hopkins et al. (2015) that estimates bloom start and end dates from [PIC] time series. To further improve the detection of coccolithophore blooms, we added the constraints that [PIC] exceeded 0.7 mmol m^−3^ and that %PIC exceeded 10%. This modified method compared favorably to occurrences of coccolithophore blooms reported in situ by Read et al. (2007) (see Figure S6 in the supporting information).

### 2.5 Statistical Analysis

Differences in *b*_bp_/*c*_p_ and *b*_bp_/[Chl‐*a*] between coccolithophore bloom and non‐bloom conditions were evaluated with the Kruskal‐Wallis test (i.e., one‐way ANOVA on ranks).

To detect coccolithophore blooms with BGC‐Argo floats, we set up a method based on *b*_bp_/*c*_p_ and *b*_bp_/[Chl‐*a*] thresholds derived from a Receiver Operating Characteristic (ROC) analysis (Woodward, 1953). The ROC analysis estimates the threshold value that optimally discriminates between coccolithophore bloom and non‐bloom condition by maximizing the sensitivity (i.e., the proportion of coccolithophore bloom cases correctly identified) and the specificity (i.e., the proportion of non‐bloom cases correctly identified).

## 3 Results and Discussion

### 3.1 BGC‐Argo Float Trajectories in Zones of Coccolithophore Blooms

**Figure 1:**
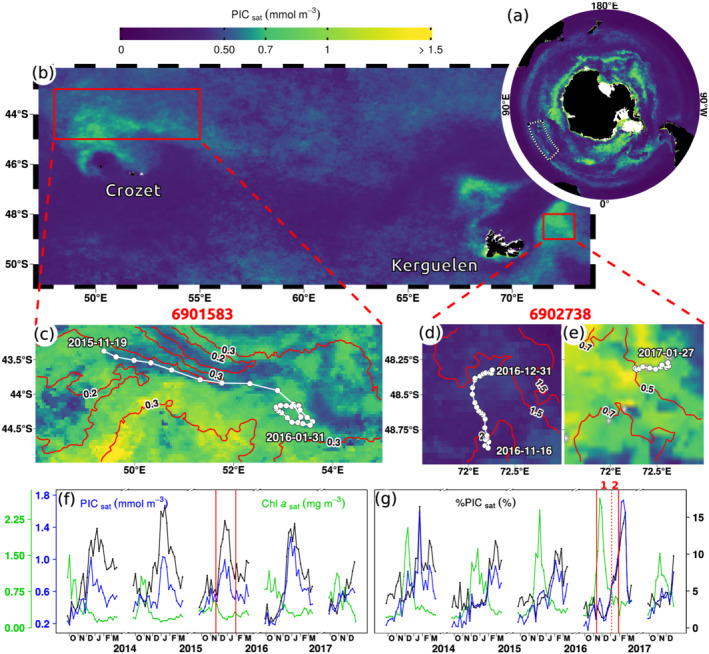
Float trajectories and coccolithophore bloom occurrences. (a) Summer climatology of satellite [PIC] (2012–2018) showing the Great Calcite Belt and (b) high‐[PIC] patches in the study area. Red rectangles outline float sampling zones. Trajectories of floats 6901583 (c) and 6902738 during (d) the Period 1 of high [Chl‐*a*] and (e) the Period 2 of increasing [PIC]. Background maps are satellite [PIC] averaged over the duration of float operations. Red lines correspond to isolines of satellite [Chl‐*a*], and concurrent [Chl‐*a*] maps are displayed on Figure S8 in the supporting information. (f, g) Area‐averaged time series of [PIC], [Chl‐*a*] and %PIC for both sampling zones, i.e., (f) is the time series for the area shown in (c) and (g) for (d)/(e). Vertical red lines indicate periods sampled by floats, and the dashed line separates Periods 1 and 2.

**Figure 2:**
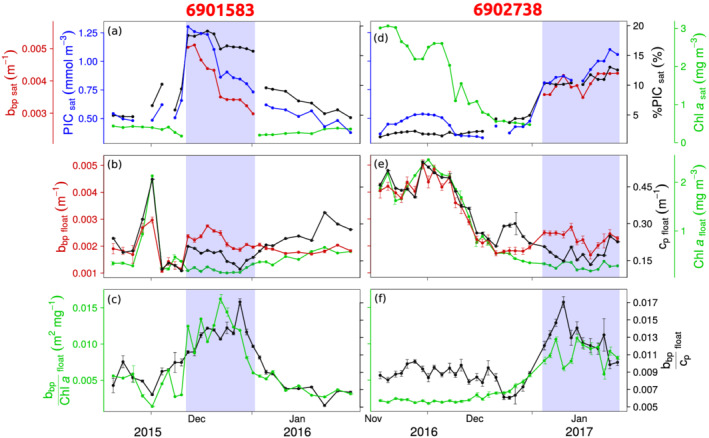
Optical measurements and satellite products during the operation of float 6901583 (a–c) and 6902738 (d–f). Time series of (a,d) [PIC], %PIC, [Chl‐*a*], and *b*_bp_ measured by satellites; (b,e) *b*_bp_, *c*_p_, and [Chl‐*a*] measured by floats; (c,f) *b*_bp_/[Chl‐*a*] and *b*_bp_/*c*_p_ measured by floats. Blue bands delineate the coccolithophore bloom period identified by the satellite detection method. Error bars indicate the standard deviation from the surface average.

Both floats operated in the Great Calcite Belt, a large band of elevated [PIC] in the Southern Ocean in Austral summer (Figure 1a) resulting from coccolithophore blooms (Balch et al., 2011, 2016; Garcia et al., 2011; Holligan et al., 2010). Float 6901583 drifted north of Crozet Islands and float 6902738 drifted east of Kerguelen Islands. Both zones have high summer [PIC] (Figures 1b, 1f, and 1g) caused by coccolithophore blooms dominated by *E. huxleyi* (Mohan et al., 2008; Patil et al., 2017; Read et al., 2007; Rembauville et al., 2016), giving the milky‐turquoise ocean‐color characteristic during float sampling (Figure S7 in the supporting information). Float 6901583 drifted on the northern edge of a coccolithophore bloom (Figure 1c), and float 6902738 near the core of a coccolithophore bloom (Figure 1e). The coccolithophore bloom detection method proposed in this study from time series analyses of satellite [PIC] showed that both floats operated throughout a coccolithophore bloom (Figures 2a and 2d).

Float 6902738 also sampled a bloom of non‐calcifying phytoplankton, as evidenced from the high [Chl‐*a*] values observed from space (Figures 1d and 2d) and by the float (Figure 2e). This pattern, of successive blooms of non‐calcifying and calcifying phytoplankton throughout Austral spring and summer, appears to repeat itself on an annual basis as observed from ocean‐color satellites time series from recent years (Figure 1g). Ship‐based work in this area also confirmed a successional pattern, with a diatom bloom preceding an *E. huxleyi* bloom (Rembauville et al., 2016).

### 3.2 BGC‐Argo Float Data in Coccolithophore Blooms

Both floats captured the evolution of a summer coccolithophore bloom, evidenced by satellite [PIC] that perceive changes in coccolith concentrations in accordance with satellite *b*_bp_ (*r* ^2^ = 0.82 between [PIC] and *b*_bp_ inside coccolithophore blooms on Figures 2a and 2d). Compared to coccolithophore blooms along the Patagonian Shelf (Garcia et al., 2011) or in the Subpolar Atlantic (Balch et al., 1996) where [PIC] can reach over 10 mmol m^−3^, the coccolithophore blooms sampled in this study are of moderate intensity, as revealed by average [PIC] of 0.97 ± 0.17 mmol m^−3^ (Figures 2a and 2d).

The coccolithophore bloom period coincides with elevated values of *b*_bp_/[Chl‐*a*] and *b*_bp_/*c*_p_ measured by floats (Figures 2c and 2f), compared to values outside the bloom period. The mean value of the *b*_bp_/*c*_p_ ratio in coccolithophore blooms equalled 0.013 ± 0.002, which is close to the value of 0.01 reported by Balch et al. (1996), and significantly higher than the value of 0.008 ± 0.001 outside coccolithophore blooms (*p* < 10^−2^). The bulk refractive index retrieved from the mean *b*_bp_/*c*_p_ (ratio as a proxy for the backscattering ratio) in coccolithophore blooms equalled 1.13 (Twardowski et al., 2001), which is intermediate between a pure population of phytoplankton cells (1.05: Bricaud et al., 1988) and calcite particles (1.2: Lide, 1997). Furthermore, the mean *b*_bp_/[Chl‐*a*] ratio in coccolithophore blooms sampled by floats 6901583 and 6902738 was 0.012 ± 0.003 and 0.009 ± 0.002 m^2^ mg^−1^, respectively. These values in coccolithophore blooms are roughly three times higher than the corresponding mean values outside coccolithophore blooms, respectively, 0.004 ± 0.001 and 0.003 ± 0.001 m^2^ mg^−1^ (*p* < 10^−2^). These observations are consistent with increased backscattering owing to non‐chlorophyllous, high‐refractive‐index particles, such as coccoliths. Both optical ratios, *b*_bp_/*c*_p_ and *b*_bp_/[Chl‐*a*], thus allowed successful discrimination of coccolithophore blooms (Figures 2c and 2f), even in these blooms of moderate intensity.

**Figure 3:**
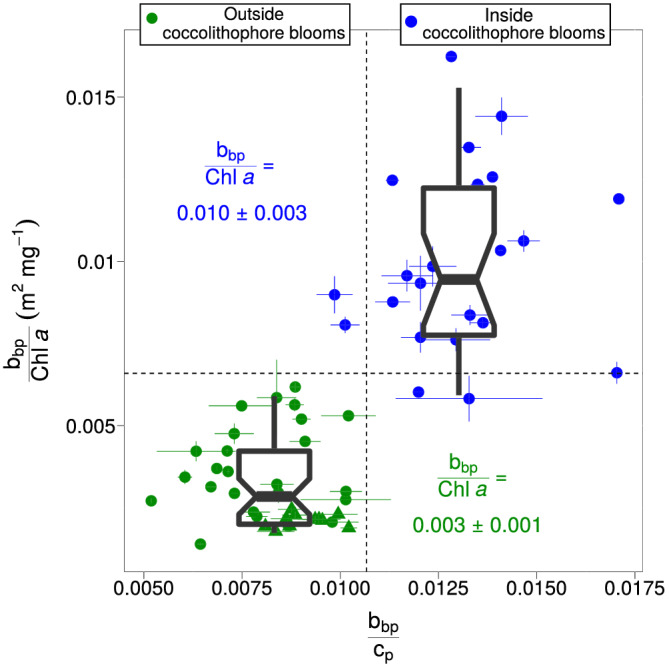
Optical ratios inside (blue dots) and outside (green dots) coccolithophore blooms sampled by floats 6901583 and 6902738. The dashed lines are the *b*_bp_/*c*_p_ and *b*_bp_/[Chl‐*a*] thresholds 0.011 and 0.007 m^2^ mg^−1^, respectively, that best discriminate coccolithophore blooms from non‐bloom cases.

ROC analyses revealed threshold values for the *b*_bp_/*c*_p_ and *b*_bp_/[Chl‐*a*] ratios of 0.011 and 0.007 m^2^ mg^−1^, respectively, that allowed optimal discrimination between coccolithophore bloom and non‐bloom conditions (Figure 3). Each threshold correctly identified 91% of coccolithophore bloom and 100% of non‐bloom cases. Discrimination between bloom and non‐bloom conditions using either the *b*_bp_/*c*_p_ or the *b*_bp_/[Chl‐*a*] ratio thus rendered similar statistics for sensitivity and specificity. This offers the possibility to extend our analysis to the global scale, as all BGC‐Argo floats are capable of measuring the *b*_bp_/[Chl‐*a*] ratio while only few floats measure *c*_p_.

### 3.3 Toward a Global Detection of Coccolithophore Blooms With BGC‐Argo Floats

**Figure 4:**
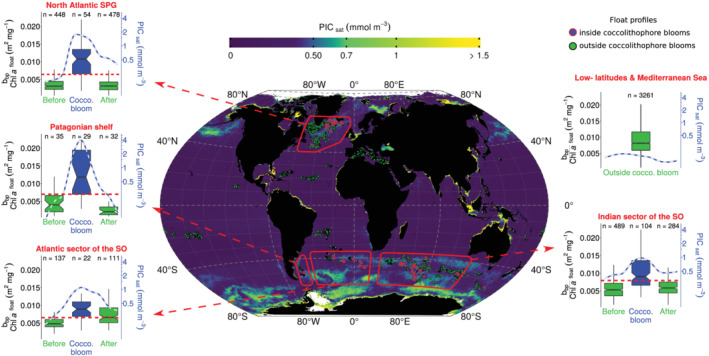
Detection of coccolithophore blooms with *b*_bp_/[Chl‐*a*] thresholds. The map reveals locations of profiles inside coccolithophore blooms detected by satellites. The background map is the summer climatology (2012–2018) of satellite [PIC] for each hemisphere. Boxplots show distributions of *b*_bp_/[Chl‐*a*] before, during, and after coccolithophore blooms in four temperate and subpolar regions identified with red polygons on the map, and in low‐latitudes (i.e., <35°) and Mediterranean Sea where no coccolithophore bloom was detected. Horizontal red dashed lines are the *b*_bp_/[Chl‐*a*] thresholds reported in Table 1. Blue lines are [PIC].

Using global observations of satellite [PIC], we identified 209 float profiles inside coccolithophore blooms and 5,330 outside blooms (Figure 4). Profiles associated with coccolithophore blooms were systematically recorded in temperate and subpolar waters (i.e., Southern Ocean and North Atlantic subpolar gyre), where *E. huxleyi* generally dominates coccolithophore populations (Balch et al., 1996, 2019; Patil et al., 2017; Poulton et al., 2011). In those waters, a regional analysis of the *b*_bp_/[Chl‐*a*] ratio revealed significantly higher values inside coccolithophore blooms (*p* < 10^−5^), consistent with observations from the two floats in the Southern Ocean (section 3.2). Thresholds for *b*_bp_/[Chl‐*a*] ranged from 0.0063 m^2^ mg^−1^ in the North Atlantic subpolar gyre to 0.0078 m^2^ mg^−1^ in the Indian sector of the Southern Ocean (Table 1). Regional differences in *b*_bp_/[Chl‐*a*] thresholds can be expected owing to differences in backscattering properties of coccolithophore blooms (e.g., the ratio of free to attached coccoliths, Balch et al., 1999, or the morphotype of coccoliths, Neukermans & Fournier, 2018), in regional variability in background *b*_bp_ associated with non‐algal material (Bellacicco et al., 2019; Zhang et al., 2020), or in phytoplankton community composition and physiology (Barbieux et al., 2018; Cetinić et al., 2015).

**Table 1:** List of *b*_bp_/[Chl‐*a*] and *b*_bp_ Thresholds for Temperate and Subpolar Regions and Their Performances to Detect Coccolithophore Blooms (i.e., Sensitivity and Specificity)

Regions	Thresholds		Performances (%)	
	*b*_bp_/[Chl‐*a*] (m^2^ mg^−1^)	*b*_bp_ (m^−1^)	Sensitivity	Specificity
North Atlantic subpolar gyre	0.0063	0.0033	74	99
Patagonian shelf	0.0069	0.0035	72	100
Indian sector of the Southern Ocean	0.0078	0.0019	60	98
Atlantic sector of the Southern Ocean	0.0064	0.0021	73	94
Total			67	98

Occasionally, high values of *b*_bp_/[Chl‐*a*] are found in waters where no coccolithophore blooms were detected, such as the Mediterranean Sea and low‐latitude waters (latitudes <35°). In these waters, elevated values of *b*_bp_/[Chl‐*a*] were generally associated with low values of [Chl‐*a*], rather than high values of *b*_bp_, typical for coccolithophore blooms (Figure S9). We therefore improved the accuracy of the bloom detection method based on *b*_bp_/[Chl‐*a*] by adding thresholds on *b*_bp_, derived from ROC analysis on the profiles with elevated *b*_bp_/[Chl‐*a*] (i.e., *b*_bp_/[Chl‐*a*] above thresholds).

Combining *b*_bp_/[Chl‐*a*] and *b*_bp_ thresholds allows the method to detect the predominance of non‐chlorophyllous high‐refractive‐index particles that are abundant enough to intensely backscatter the light, which are conditions reported in coccolithophore blooms of *E. huxleyi* identified here by satellite [PIC]. With the *b*_bp_/[Chl‐*a*] and *b*_bp_ thresholds listed on Table 1, the method successfully identified nearly three‐quarter of coccolithophore bloom cases in all temperate and subpolar regions except in the Indian sector of the Southern Ocean, and greatly differentiated bloom from non‐bloom cases as illustrated by high values of specificity (Table 1). The combination of *b*_bp_/[Chl‐*a*] and *b*_bp_, acquired by all BGC‐Argo floats, thus allows the effective detection of coccolithophore blooms in situ at the global scale.

## 4 Conclusions

In this study, ocean‐color satellite observations of coccolithophore blooms were matched with bio‐optical measurements from BGC‐Argo floats to test the feasibility of developing a float‐based detection method for coccolithophore blooms. A spatiotemporal analysis of bio‐optical observations from two floats in the Southern Ocean reveal significant increases in both *b*_bp_/*c*_p_ and *b*_bp_/[Chl‐*a*] in coccolithophore blooms. These increases are consistent with accumulations of coccoliths; non‐chlorophyllous, high‐refractive‐index particles, typically associated with coccolithophore blooms of *E. huxleyi*. Since only a limited number of BGC‐Argo floats measure *c*_p_, we developed a detection method based on measurements of *b*_bp_ and [Chl‐*a*] alone, which are measured by all BGC‐Argo floats with global distribution. We conclude that coccolithophore blooms can be successfully identified from floats using regional thresholds on *b*_bp_/[Chl‐*a*] and *b*_bp_. This opens perspectives for global‐scale studies of coccolithophore blooms with the global array of BGC‐Argo floats, expected to expand to thousand floats in the near future (Claustre et al., 2020). Whereas ocean‐color satellites offer global‐scale observations of coccolithophore blooms in the surface ocean in cloud‐free conditions, floats extend our view of carbon particles down to 1,000 m deep, complementing the observational dimensions covered by satellites. Future work will include observations from the subsurface ocean accessible by these floats, which offer the potential to investigate the global impact of coccolithophore blooms on ocean biogeochemistry as their role in sinking carbon to the deep ocean.

## Conflict of Interest

The authors declare no conflict of interest.

## Erratum

In the originally published version of this article, links to the data for BGC‐Argo did not have a FAIR‐compliant DOI. This error has since been corrected, and the present version may be considered the authoritative version of record.

## Supporting information

Supporting Information S1

## Data Availability

The data supporting the conclusions of this study are freely available at https://doi.org/10.17882/76521, and all BGC‐Argo data are available at https://doi.org/10.17882/42182#71394 or ftp://ftp.ifremer.fr/ifremer/argo/dac/. These data were collected and made freely available by the International Argo Program and the national programs that contribute to it (http://www.argo.ucsd.edu, http://argo.jcommops.org).
