# Supplementary material for: Detection of Coccolithophore Blooms With BioGeoChemical‐Argo Floats
Source: Geophys Res Lett. 2020 Nov 25;47(23):e2020GL090559. doi: 10.1029/2020GL090559 (PMC7757229; doi:10.1029/2020GL090559)
Supplement: Supplementary file 1 — Supporting Information S1 [file GRL-47-e2020GL090559-s001.docx]

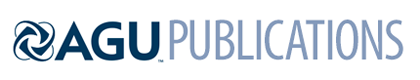


*Geophysical Research Letter*

Supporting Information for

**Detection of coccolithophore blooms with BioGeoChemical-Argo floats**

L. Terrats^1,2^, H. Claustre^1^, M. Cornec^1^, A. Mangin^2^ and G. Neukermans^3,4^

^1^ Sorbonne Université, CNRS, Laboratoire d'Océanographie de Villefranche, LOV, F-06230, Villefranche-sur-Mer, France

^2^ ACRI-ST, F-06904 Sophia Antipolis, France

^3^ Ghent University, Biology Department, MarSens Research Group, Krijgslaan 281 – S8, 9000 Ghent, Belgium

^4^ Flanders Marine Institute (VLIZ), InnovOcean site, Wandelaarkaai 7, 8400, Ostend, Belgium

**Contents of this file**

Text S1 to S4

Figures S1 to S9

Table S1

**Introduction**

This supporting information:

- informs of the sensitivity of surface c_p_ to the different b_bp_/c_p_ values which are used to correct the drift over time (**Text S1** and **Table S1**).
- details how we corrected float FChl-*a* for NPQ (**Text S2** and **Figure S1 and S2)** and how we converted FChl-*a* to [Chl-*a*] (**Text S3)**.
- explains the choice of the 9-day temporal window to matchup satellite and float data (**Text S4 and Figures S3, S4, and S5)**.
- compares the satellite detection of coccolithophore blooms with *in-situ* data (**Figure S6**).
- provides the true-colour satellite images and the [Chl-*a*] maps of the areas sampled by floats 6901583 and 6902738 (**Figure S7 and S8 respectively**).
- Shows the distribution of b_bp_ that help identifying coccolithophore blooms in complement to b_bp_/[Chl-*a*] (**Figure S9**).

**Text S1: Correction of the drift.**

Correcting the c_p_ profiles for the drift is commonly performed by assuming a null c_p_ value at 1000m (Bishop & Wood, 2009; Xing et al., 2014). However, a significant number of particles is measured at 1000m (Poteau et al., 2017) so we corrected cp drift using b_bp_ values at 1000m and assuming a constant b_bp_/c_p_ ratio.

Remineralization and fragmentation processes in the twilight zone control the vertical flux of particles and typically result in deep (1000m) particle populations dominated by non-chlorophyllous refractory material with a high bulk refractive index. For such particle populations the b_bp_/c_p_ ratio ranges from 1% to 3% (Chami et al., 2005). Our results indicate that the choice of the b_bp_/c_p_ ratio at 1000 m in the range 1–3% did not significantly influence surface c_p_ values (Table S1). Therefore, we chose the median value of 2% as the constant b_bp_/c_p_ ratio at 1000m.


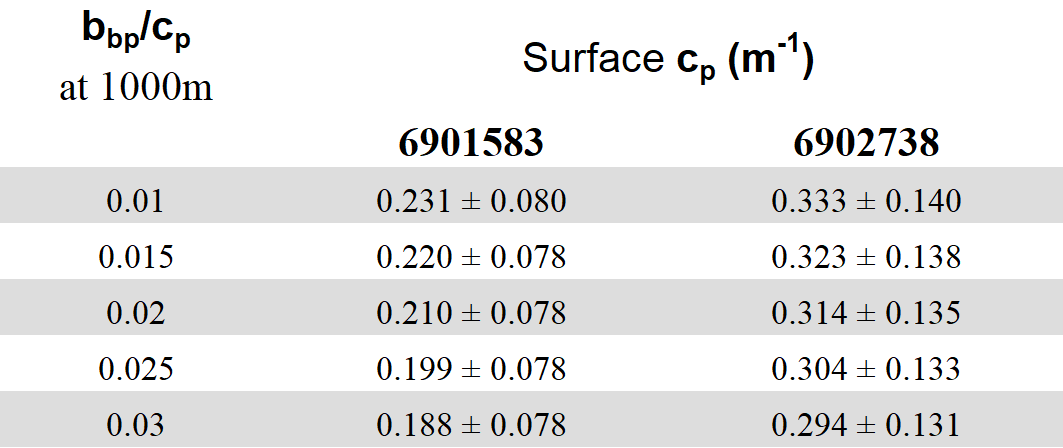


**Table S1.** Impact of the choice of b_bp_/c_p_ at 1000 m for the retrieval of c_p_ at surface (mean ± standard deviation).

**Text S2: Correction of the Non-Photochemical Quenching (NPQ).**

The NPQ correction method of Xing *e**t* *a**l**.*, (2018), called X18, is the most accurate to date. We assessed its performances at surface (i.e. 0-15 m) by comparing day- and night-time [Chl-*a*] that we paired following these criteria : 1) day- and night-time profiles are within a time window of 24h; 2) the day- and night-time b_bp_ profiles differ by less than 30% inside the mixed layer (percentage estimated with the Mean Absolute Percentage Difference, as in Xing *e**t* *a**l**.*, (2018); 3) the day- and night-time [Chl-*a*] profiles differs by less than 30% at depth where NPQ is absent, i.e. where photosynthetically active radiation is below 15 µmol quanta m^-2^ s^-1^ (Xing et al., 2018); 4) the Mixed Layer Depth (MLD) differs by less than 15m between day- and night-time profiles. This dataset provided 98 pairs of profiles, from which we derived the error defined as the relative difference between day- and night-time [Chl-*a*] at surface.

The X18 method corrects differently according to the status of water column stratification either deep-mixing conditions where NPQ only affects the top of the mixed layer, or shallow-mixing conditions where NPQ affects the whole mixed layer and potentially below. For deep-mixing cases (n = 59 pairs, MLD = 78 ± 48 m), NPQ is corrected by multiplying b_bp_ with a [Chl-*a*]/b_bp_ ratio determined inside the mixed layer. This method originating from Sackmann *e**t* *a**l**.*, (2008) assumes uniformity in particle composition inside the mixed layer. For shallow-mixing cases (n = 39 pairs, MLD = 38 ± 24 m), an empirical sigmoid function corrects for NPQ. This correction sometimes retrieves surface [Chl-*a*] that differs from the night measurements by more than 200 % (Figure S1). We explain such differences at surface by wrong estimates from the empirical function rather than biological processes, since b_bp_ did not covary with [Chl-*a*] (Figure S1b).

We modified the method for shallow-mixing cases as follows. Below MLD, we kept correcting NPQ with the sigmoid function of the X18 method and we computed the corrected ratio of [Chl-*a*]/b_bp_ at MLD. Above MLD, we multiplied b_bp_ by the [Chl-*a*]/b_bp_ ratio at MLD, assuming a constant [Chl-*a*]/b_bp_ ratio inside the remaining upper part of the mixed layer. This method, called X18_S08, showed better performances for shallow-mixing profiles (Figures S1c and S1a) and a better consistency as we assumed the uniformity in particle composition in both shallow- and deep-mixing cases.

We assessed the variation of [Chl-*a*]/b_bp_ in the mixed layer using night measurements unbiased by NPQ. Most [Chl-*a*]/b_bp_ values in the mixed layer differed by less than 15% from the ratio at MLD and showed no bias as the median differences were confined around 0 (figure S2), thus demonstrating the uniformity of [Chl-*a*]/b_bp_ inside the mixed layer. This supports the key assumption and the use of the S08_X18 method to correct NPQ the most accurately.


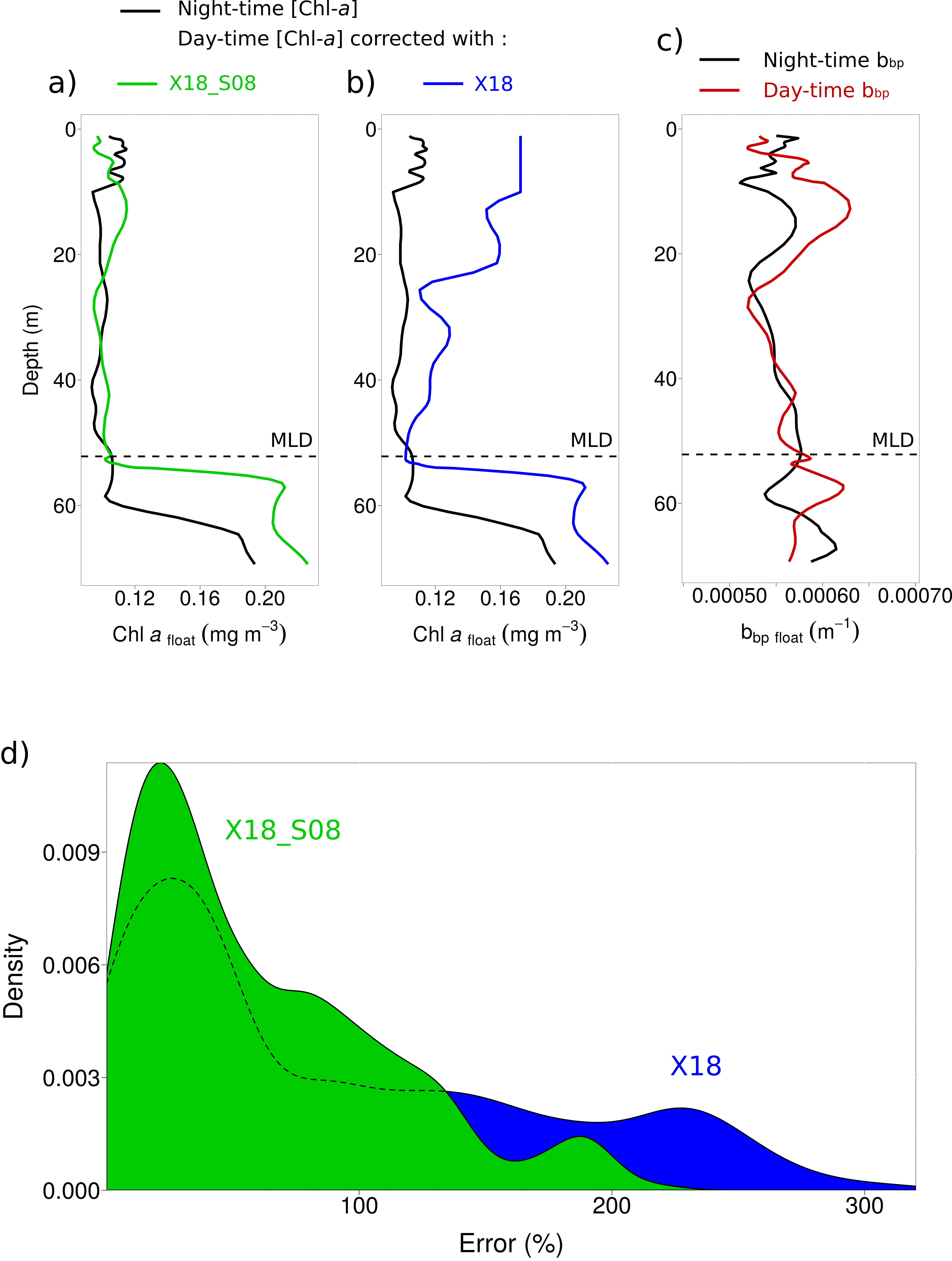
**Figure S1. Performances of the X18 and X18_S08 methods** to correct shallow-mixing profiles for NPQ. Profiles of night-time [Chl-*a*] (black line) and day-time [Chl-*a*] corrected for NPQ with a) X18_S08 (green line) and b) X18 (blue line). c) Profiles of day-time (red line) and night-time (black line) b_bp_. d) Distribution of error values retrieved at surface (0-15 m) for methods X18_S08 (green) and X18 (blue).


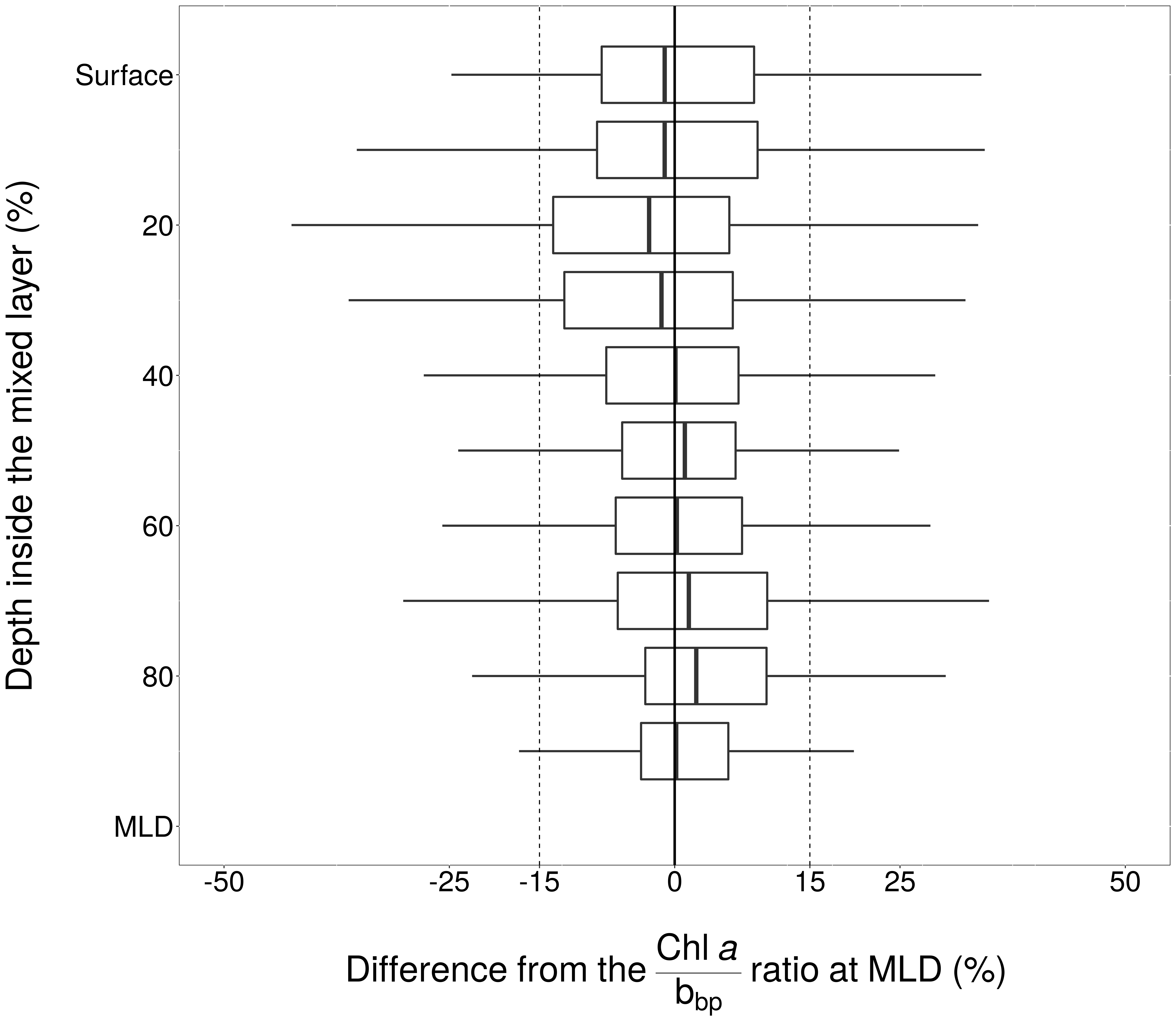
**Figure S2.** Vertical distribution of differences between [Chl-*a*]/b_bp_ values at MLD and inside the mixed layer (%).

**Text S3: Converting float FChl-*a* to [Chl-*a*]**

We converted float FChl-*a* to [Chl-*a*] using a factory calibration coefficient which is known to overestimate *in-situ* [Chl-*a*] and hence require adjustments (Roesler et al., 2017). Roesler *e**t* *a**l**.*, (2017) adjusted the factory’s coefficient with factors ranging from 1 to 6 according to location, which demonstrates the significance of a regional adjustment to accurately convert FChl-*a* to [Chl-*a*]. Such factors remain unknown for some of the areas investigated in the present study (e.g. subtropical gyres), but we set up a method that circumvents this issue and subsequently adjusted [Chl-*a*] for all floats.

We adjusted float [Chl-*a*] with matchups between satellite and float [Chl-*a*] using a temporal window of 1-day to balance the quantity and accuracy of matchups in high-latitude waters (Haëntjens *e**t* *a**l**.*, 2017). For each float totalizing at least 15 matchups, we computed a linear regression between float and satellite [Chl-*a*] and defined the significant slope (p < 0.05) as the factor to adjust [Chl-*a*]. When the correction factor could not be computed, either because of too few matchups or a non-significant slope, the float data were removed from the study. This method adjusts for [Chl-*a*] on a float-by-float basis, thus accounting for regional variations required to accurately estimate [Chl-*a*].

In high-latitude waters, the correction factors are similar to the ones of Roesler *e**t* *a**l.*, (2017)’s, with 4.4 ± 1.1 (n = 18 floats) compared to 4.95 in the Southern Ocean and 3.5 ± 1.2 (n = 12 floats) compared to 3.38 in the North Atlantic subpolar gyre. In the Mediterranean Sea and low-latitudes waters (i.e. < 35°), we retrieved 3.1 ± 1.7 (n = 58 floats), with specific values of 2.44 ± 0.6 (n = 34 floats) in the Mediterranean sea as compared to 1.66 for Roesler *e**t* *a**l**.*, (2017). Since we retrieved similar factors to Roesler *e**t* *a**l**.*, (2017)’s in most regions, we concluded that the satellite-based adjustment method, applicable on every float, tends to reliably adjust [Chl-*a*].

**Text S4: The temporal window of matchups.**

Our purpose of matching satellite and *in-situ* data was not to validate ocean-colour sensors nor products, but rather to obtain a continuous gap-free time series of satellite data corresponding to the float trajectory (which is particularly challenging in cloudy areas such as the Southern Ocean). Therefore, we used the GlobColour ocean-colour satellite dataset that provides merged products from all available ocean-colour satellites, binned into 4km bins. In persistently cloudy areas such as the Southern Ocean, this further implies data averaging over larger spatial and temporal scales until a gap-free time series is achieved (also noted previously by Haëntjens et al., (2017)).

We extended the spatial window to 20 x 20 km to increase the number of matchups by 13 ± 5% or 4 ± 3 % compared to the 12 x 12 km or 4 x 4 km windows, respectively (Figure S3). This window size is close to the recommendations from Haëntjens et al., (2017) to achieve matchups in the Southern Ocean using satellite data within a 16 km diameter circle around the float profile.

We assessed the performance of temporal windows larger than 1-day (i.e. 3-day, 5-day, 7-day, and 9-day) with three metrics: the coverage of time series, the bias, and the error from the 1-day matchups estimated with the Mean Absolute Error (MAE) (Seegers et al., 2018) defined by:

${MAE}_{xday}$ = $\frac{1}{N}$ $\times$ $\sum_{i=1}^{N} | X_{xday}-X_{1day}|$

with *xday* a temporal window larger than 1-day. Only the [PIC] and %PIC parameters were considered as they are the most relevant satellite variables for this study (i.e. they describe the dynamic of coccolithophore blooms).

The 9-day temporal window provides the most of matchups (i.e. 91%) characterized by moderate bias of +1% for [PIC] and +3.5% for %PIC but the lowest accuracies with MAE of 13% for [PIC] and 15% for %PIC (Figure S5). The shapes of times series obtained with the 9-day temporal window resembled to others (Figure S4), as such we concluded that these lowest accuracies remain negligible for the purpose of this study. We therefore matched float and satellite data with the 9-day and 20 x 20 km windows for describing the environment around the float trajectory. In this configuration, the spatiotemporal variability of satellite-derived PIC was typically within 12% ± 10 %, indicating reasonable overall heterogeneity.


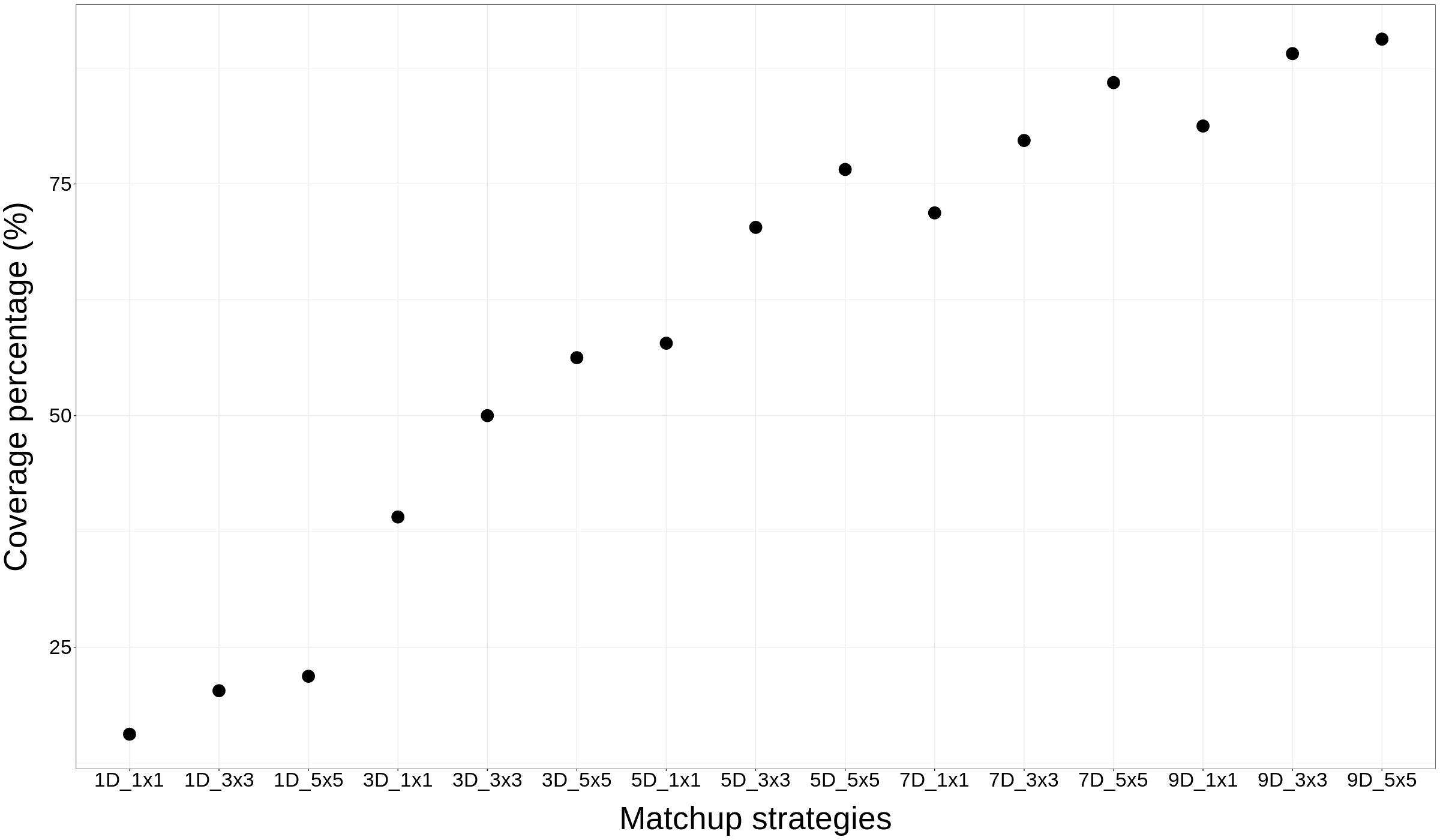


**Figure S3.** Percentages of coverage of the float time series by using different strategies of matchup. The temporal window varies from 1-day (1D) to 9-days (9D) and the spatial window from 1 x 1 (4 x 4 km) to 5 x 5 (20 x 20 km) pixel box.


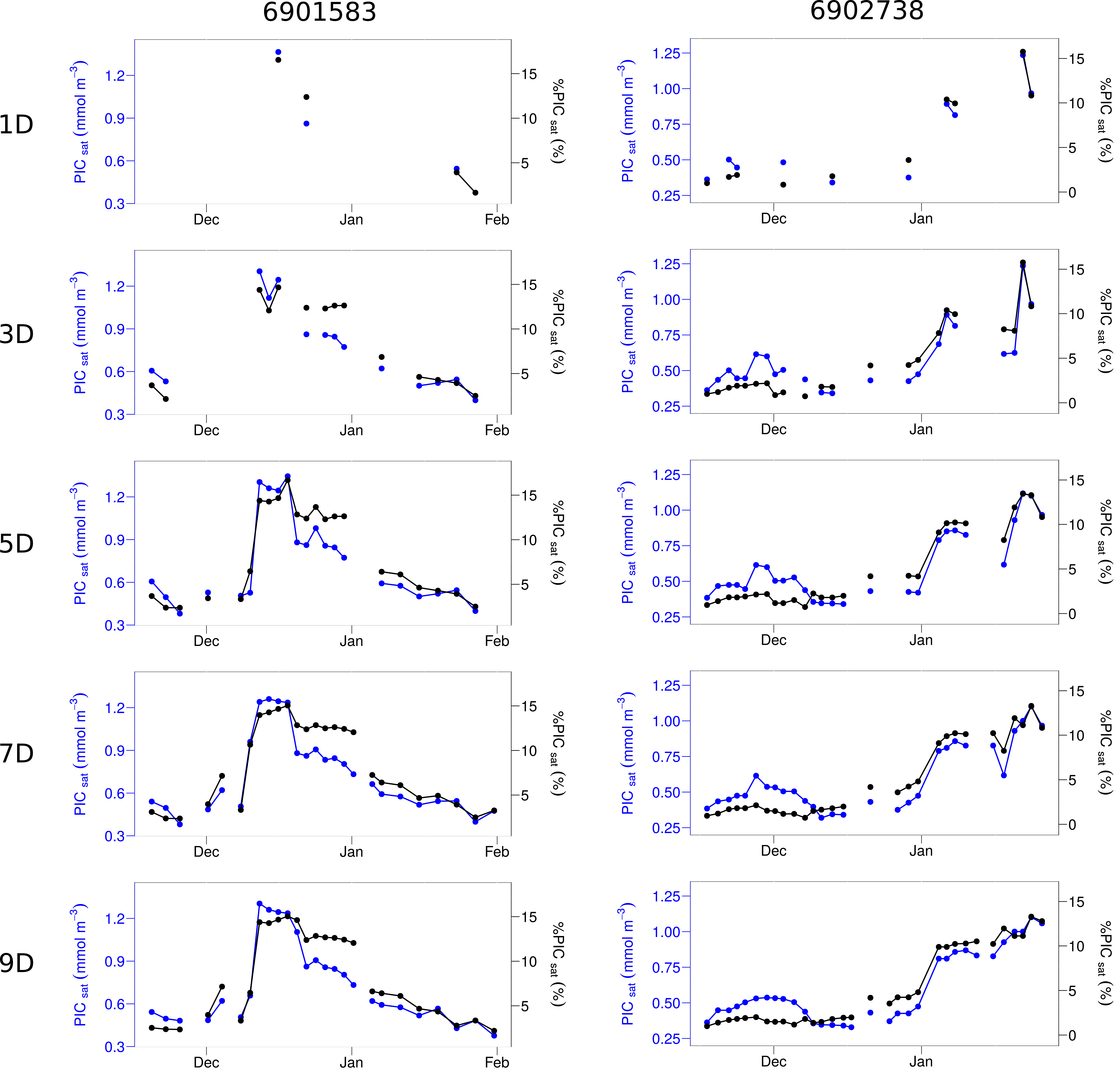


**Figure S4.** Time series of satellite [PIC] (blue) and %PIC (black) matchups for floats 6901583 (left column) and 6902738 (right column). The temporal window of matchups varies from one day (1D, on the top) to nine days (9D, on the bottom).


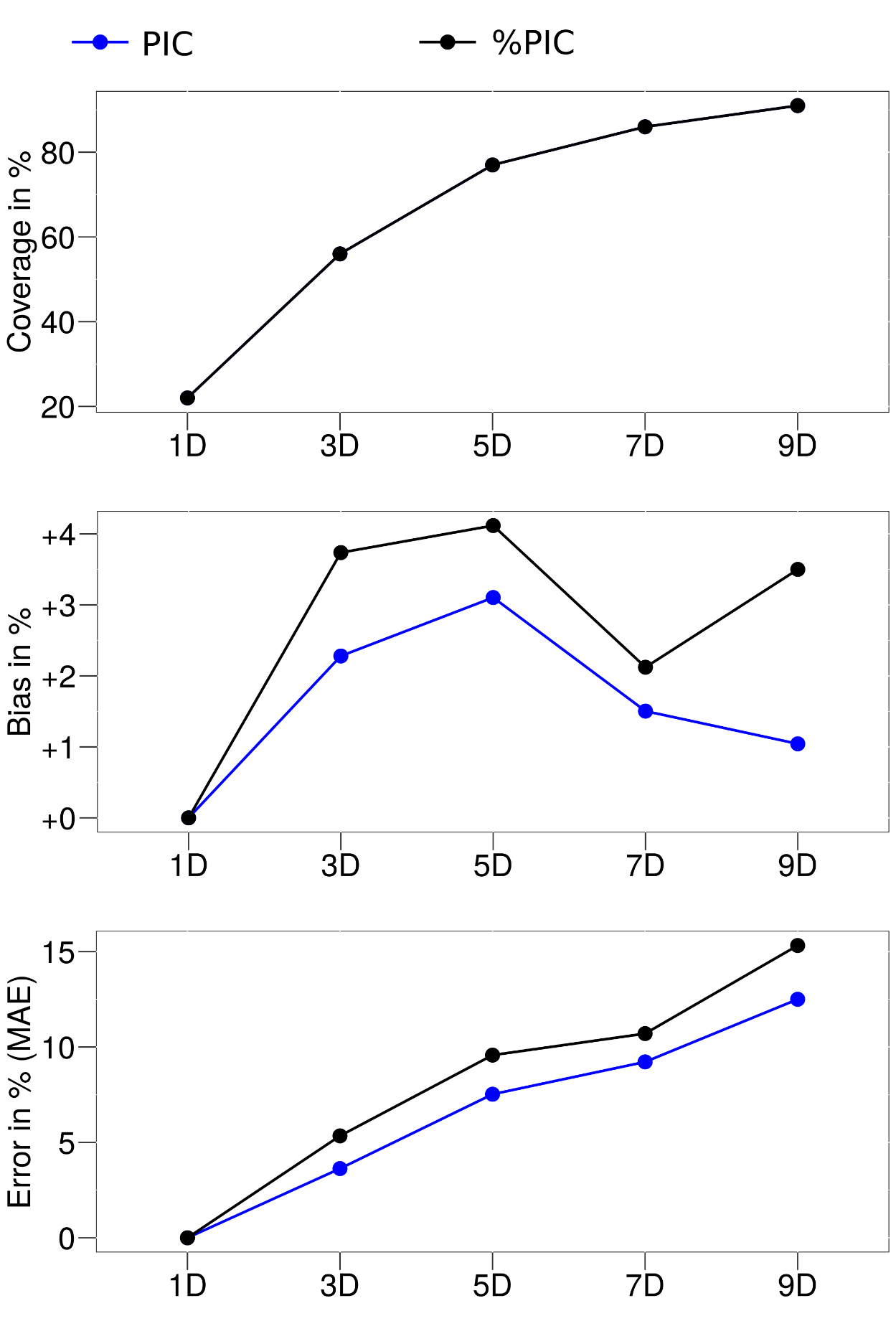


**Figure S5.** Performances of various temporal windows examined to matchup satellite [PIC] (blue) and %PIC (black) with profile locations. Performances were assessed with three metrics: a) the coverage of time series ([PIC] and %PIC are superimposed), b) the bias, c) and the MAE. Temporal windows vary from one day (1D) to nine days (9D).

**
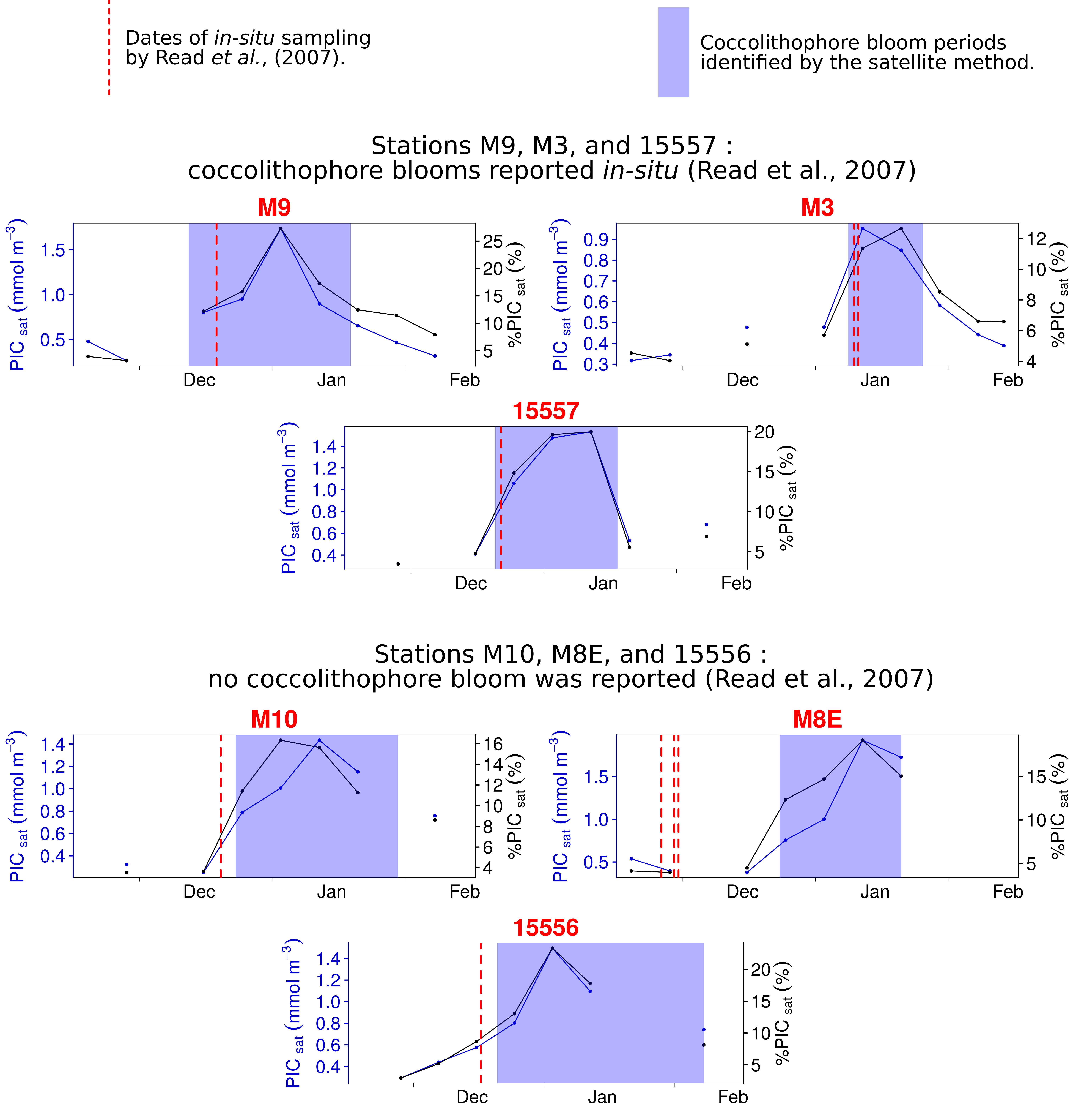
**

**Figure S6.** Detection of coccolithophore blooms with satellite and *in-situ* data. Blue and black lines are respectively satellite time series of [PIC] and %PIC at stations sampled by Read *e**t* *a**l**.*, (2007). Blue bands delineate the coccolithophore bloom period identified by the satellite method. Vertical red lines indicate the dates of *in-situ* sampling. Both *in-situ* data and satellite method agree on the presence (M9, M3, 15557) or absence (M10, M8E, and 15556) of coccolithophore blooms.


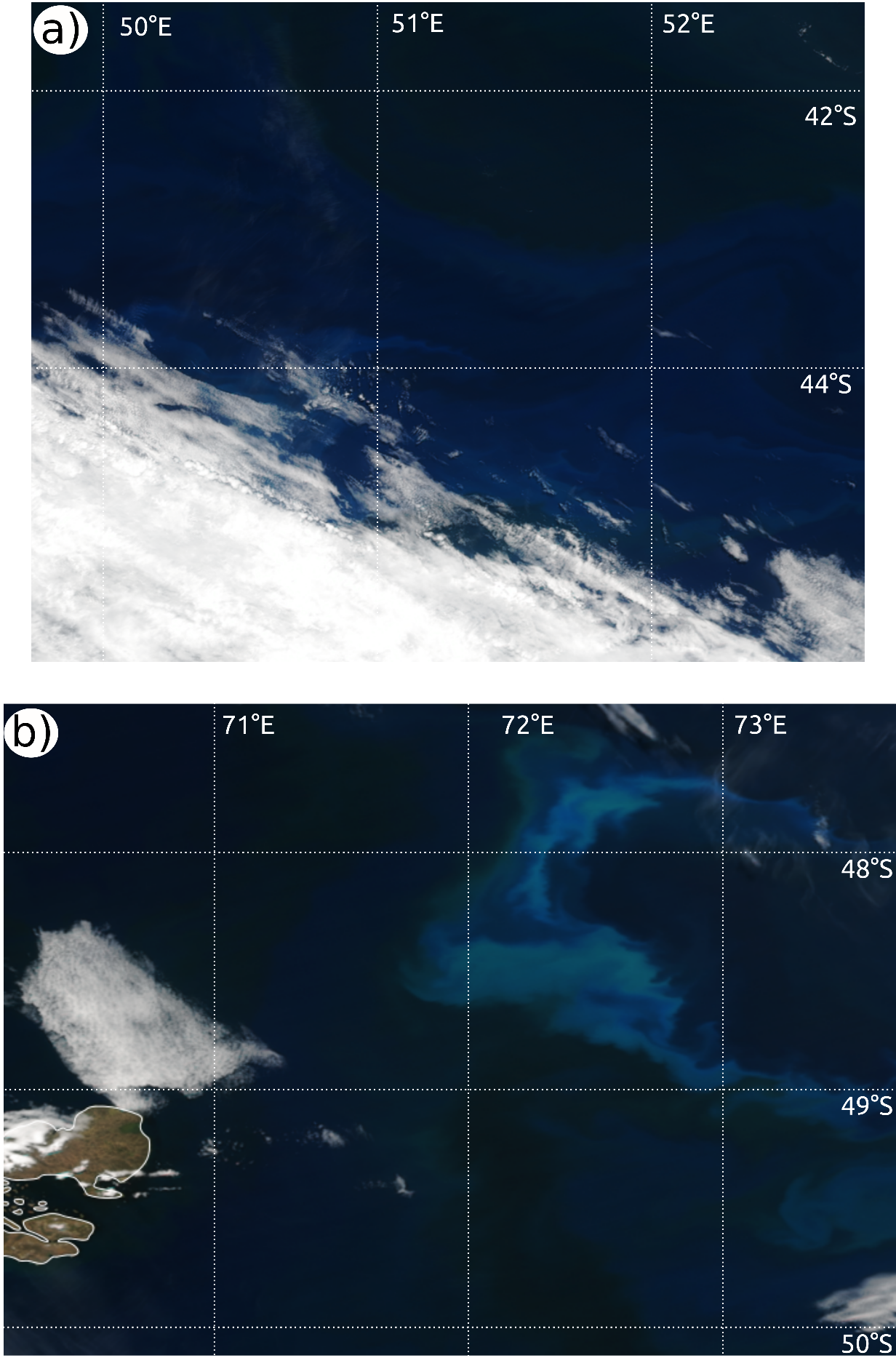


**Figure S7.** True-colour MODIS-Aqua images of the areas sampled during operations of floats a) 6901583 and b) 6902738. Image a) is acquired on December, 28, 2015 and image b) on February, 09, 2017.


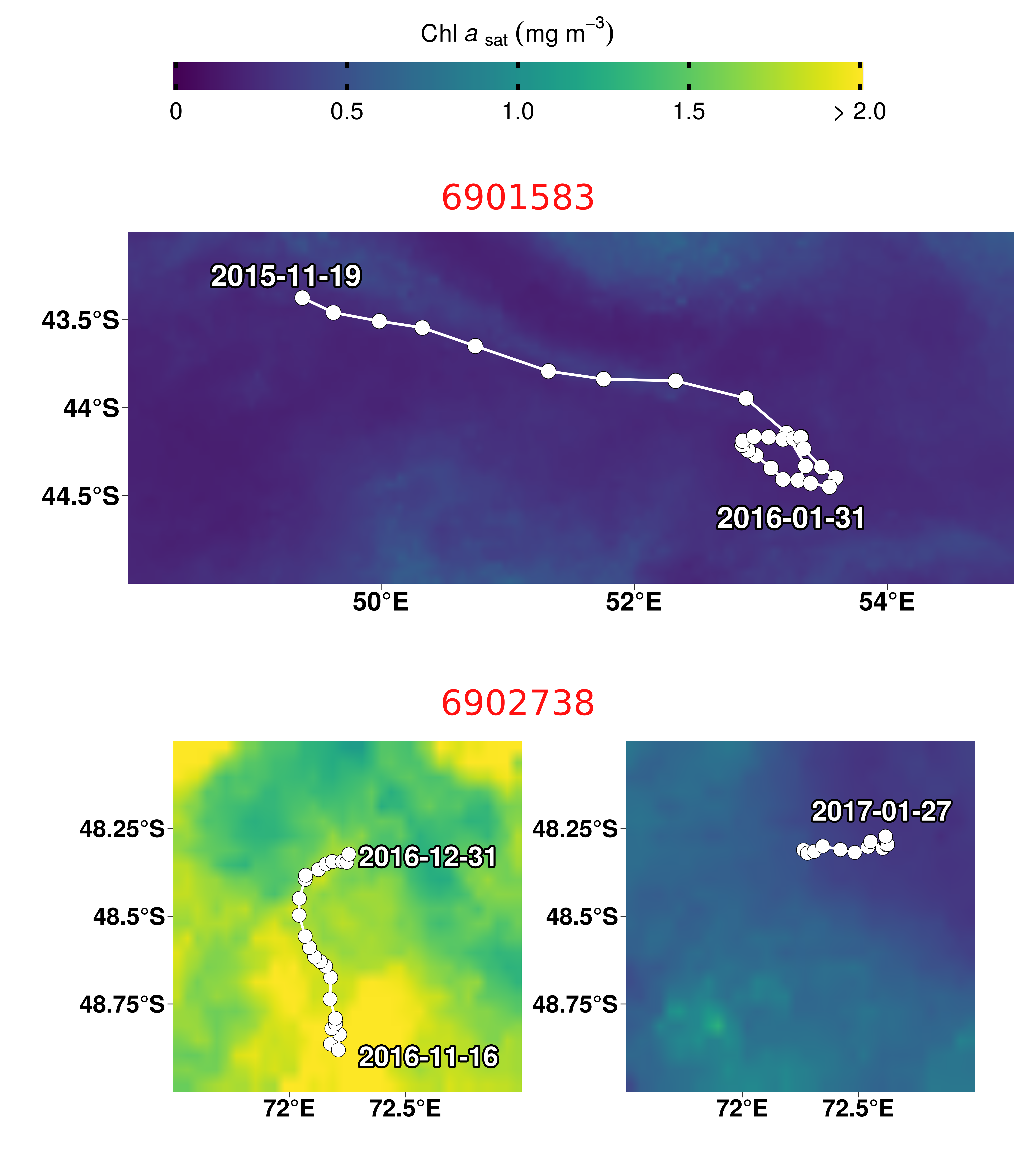


**Figure S8.** Maps of satellite [Chl-*a*] averaged over the duration of float operations.


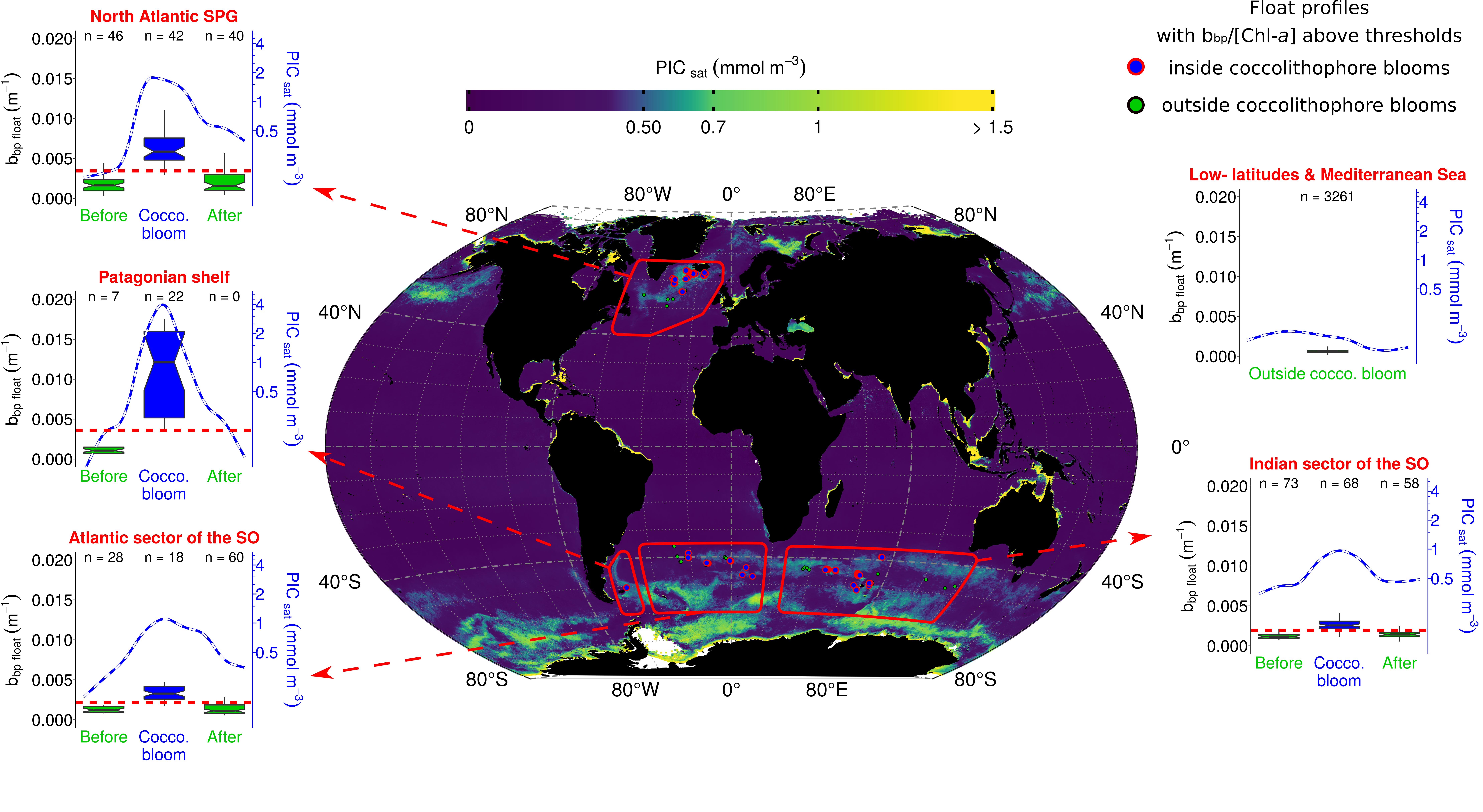
**Figure S9. Determination of b_bp_ thresholds to refine the detection method with b_bp_/[Chl-*a*].**

The map reveals locations of profiles with surface b_bp_/[Chl-*a*] above thresholds listed on Table 1, except for Mediterranean Sea & low-latitude (i.e. < 35°) regions where all profiles were reported. The background map is the summer climatology (2012-2018) of satellite [PIC] for each hemisphere. Boxplots show distributions of b_bp_ before, during, and after coccolithophore blooms in four high-latitude regions identified with red polygons on the map, and in low-latitudes and Mediterranean Sea where no coccolithophore bloom was detected. Horizontal red dashed lines are the b_bp_ thresholds (see Table 1 in the main text). Blue lines are [PIC].

**References**

Bishop, J. K. B., & Wood, T. J. (2009). Year-round observations of carbon biomass and flux variability in the Southern Ocean. *Global Biogeochemical Cycles*, *23*(2), 1–12. https://doi.org/10.1029/2008GB003206

Chami, M., Shybanov, E. B., Churilova, T. Y., Khomenko, G. A., Lee, M. E.-G., Martynov, O. V, et al. (2005). Optical properties of the particles in the Crimea coastal waters (Black Sea). *Journal of Geophysical Research: Oceans*, *110*(C11). https://doi.org/10.1029/2005JC003008

Haëntjens, N., Boss, E., & Talley, L. D. (2017). Revisiting Ocean Color algorithms for chlorophyll a and particulate organic carbon in the Southern Ocean using biogeochemical floats. *Journal of Geophysical Research: Oceans*, *122*(8), 6583–6593. https://doi.org/10.1002/2017JC012844

Poteau, A., Boss, E., & Claustre, H. (2017). Particulate concentration and seasonal dynamics in the mesopelagic ocean based on the backscattering coefficient measured with Biogeochemical-Argo floats. *Geophysical Research Letters*, *44*(13), 6933–6939. https://doi.org/10.1002/2017GL073949

Read, J. F., Pollard, R. T., & Allen, J. T. (2007). Sub-mesoscale structure and the development of an eddy in the Subantarctic Front north of the Crozet Islands. *Deep-Sea Research Part II: Topical Studies in Oceanography*, *54*(18–20), 1930–1948. https://doi.org/10.1016/j.dsr2.2007.06.013

Roesler, C., Uitz, J., Claustre, H., Boss, E., Xing, X., Organelli, E., et al. (2017). Recommendations for obtaining unbiased chlorophyll estimates from in situ chlorophyll fluorometers: A global analysis of WET Labs ECO sensors. *Limnology and Oceanography: Methods*, *15*(6), 572–585. https://doi.org/10.1002/lom3.10185

Sackmann, B. S., Perry, M. J., & Eriksen, C. C. (2008). Fluorescene quenching from Seaglider Seaglider observations of variability in daytime fluorescence quenching of chlorophyll-a in Northeastern Pacific coastal waters Fluorescene quenching from Seaglider. *Biogeosciences Discuss*, *5*, 2839–2865. https://doi.org/10.5194/bgd-5-2839-2008

Xing, X., Claustre, H., Uitz, J., Mignot, A., Poteau, A., & Wang, H. (2014). Seasonal variations of bio-optical properties and their interrelationships observed by Bio-Argo floats in the subpolar North Atlantic. *Journal of Geophysical Research: Oceans*. https://doi.org/10.1002/2014JC010189

Xing, X., Briggs, N., Boss, E., & Claustre, H. (2018). Improved correction for non-photochemical quenching of in situ chlorophyll fluorescence based on a synchronous irradiance profile. *Optics Express*, *26*(19), 24734. https://doi.org/10.1364/oe.26.024734
